# Biological Carbon Recovery from Sugar Refinery Washing Water into Microalgal DHA: Medium Optimization and Stress Induction

**DOI:** 10.1038/s41598-019-56406-x

**Published:** 2019-12-27

**Authors:** Myounghoon Moon, Won-Kun Park, William I. Suh, Yong Keun Chang, Bongsoo Lee

**Affiliations:** 1 Gwangju Bio/Energy R&D Center, Korea Institute of Energy Research (KIER), 25, Samso-ro 270beon-gil, Buk-gu, Gwangju 61003 Republic of Korea; 2 Department of Chemistry & Energy Engineering, Sangmyung University, 20 Hongjimun 2-gil, Jongno-gu, Seoul 03016 Republic of Korea; 3 Advanced Biomass R&D Center, Korea Advanced Institute of Science & Technology (KAIST), 291 Daehak-ro, Yuseong-gu, Daejeon 34141 Republic of Korea; 4 Department of Chemical and Biomolecular Engineering, Korea Advanced Institute of Science & Technology (KAIST), 291 Daehak-ro, Yuseong-gu, Daejeon 34141 Republic of Korea; 5 Department of Microbial and Nano Materials, College of Science and Technology, Mokwon University, 88 Doanbuk-ro, Seo-Gu, Daejeon 35349 Republic of Korea

**Keywords:** Environmental biotechnology, Applied microbiology

## Abstract

Sugar refinery washing water (SRWW) contains abundant levels of carbon sources and lower levels of contaminants than other types of wastewater, which makes it ideal for heterotrophic cultivation of microalgae. Here, carbon sources in SRWW were utilized for conversion into the form of value-added docosahexaenoic acid (DHA) using *Aurantiochytrium* sp. KRS101. Since SRWW is not a defined medium, serial optimizations were performed to maximize the biomass, lipid, and DHA yields by adjusting the nutrient (carbon, nitrogen, and phosphorus) concentrations as well as the application of salt stress. Optimum growth performance was achieved with 30% dilution of SRWW containing a total organic carbon of 95,488 mg L^−1^. Increasing the nutrient level in the medium by supplementation of 9 g L^−1^ KH_2_PO_4_ and 20 g L^−1^ yeast extract further improved the biomass yield by an additional 14%, albeit at the expense of a decrease in the lipid content. Maximum biomass, lipid, and DHA yields (22.9, 6.33, and 2.03 g L^−1^, respectively) were achieved when 35 g L^−1^ sea salt was applied on a stationary phase for osmotic stress. These results demonstrate the potential of carbon-rich sugar refinery washing water for DHA production using *Aurantiochytrium* sp. KRS101 and proper cultivation strategy.

## Introduction

Among an estimated total of over 300,000 species of microalgae^1^, numerous species have been found to have commercially desirable phenotypes including high biomass production, lipid content, and potential for value-added products^2,3^. In particular, a number of microalgal strains are capable of accumulating high levels of polyunsaturated fatty acids (PUFAs) and have been identified as a potential source of feedstock for the production of PUFAs as value-added products^4–6^. PUFAs are categorized as omega-3, omega-6, or omega-9 fatty acids depending on the position of the first double bond in relation to the methyl terminus^7^. Various types of PUFAs, such as omega-3 fatty acids including α-linolenic acid (ALA; C18:3 n-3), eicosapentaenoic acid (EPA; C20:5 n-3), and docosahexaenoic acid (DHA; C22:6 n-3) are essential fatty acids in the human diet^8^.

DHA is a key omega-3 fatty acid that has beneficial health effects, including reducing the risks of human cardiovascular diseases, cancer, schizophrenia, and Alzheimer’s disease^9^. It also plays an important role as a structural lipid in cell membranes and it is necessary for proper visual and neurological development in infants^10^. While the majority of DHA in the nutraceutical market is derived from fish oils, there has been increasing interest in the commercial production of microalgal DHA. Microalgae-derived DHA have several advantages to overcome current concerns on fish-derived DHA. First of all, because of the food chain system in the ocean, fish tend to accumulate more amount of heavy metals and other toxic chemicals than microalgae^11^. In addition, it is reported that DHA separation process from microalgae is easier than fish’s profiles. Even more, DHA originated from microalgae have better absorbing property than that from fish oil, too^12^. Furthermore, recent research indicates that the production of omega-3 lipids from fish oil presents a sustainability issue because over 85% of the world’s fisheries are subject to overfishing or are depleted^13^. However, in the case of microalgae, it could be fully produced by designed cultivation system in the industry, therefore, there are no concerns about the fluctuation in the production or depletion. Of course, major microalgae culture system using photosynthesis might have regular fluctuation caused by light/dark cycle and seasonal changes. However, the value-added product like DHA could justify its higher cost for heterotrophic production using carbon sources and it means there will be well controlled system to produce DHA compensate the demand with stable production facility.

Thus, PUFAs from oleaginous microalgae have attracted much attention as a sustainable source of essential omega-3 oils^14^. *Aurantiochytrium* sp. KRS101 is a candidate as a novel source of omega-3 fatty acids. This marine heterotroph requires conventional saccharides for carbon and energy sources^15^. Although the heterotrophic cultivation of microalgae can achieve a much higher cell density and faster growth rates compared with photoautotrophic cultivation, the cost of organic substrates (e.g. glucose) in a heterotrophic medium is much greater; the carbon source has been calculated to comprise of approximately 80% of the total cost of the growth medium^16^. To decrease the cultivation costs, alternative carbon sources have been intensively studied with respect to their conversion into fatty acids^17–19^.

Various organic wastewaters have been studied as an alternative carbon source for economic heterotrophic cultivation of microalgae. However, applications of wastewater were generally limited to chemicals or biofuel production rather than food and nutraceutical products due to the possible presence of toxic chemicals, heavy metals, as well as unsanitary nature of most wastewaters^20^. In this point of view, finding a clean and safe carbon-rich wastewater is important for the production of human-edible products. Wastewater resulting from food manufactory facilities, such as sugar refineries can be a major target source as they contain high concentrations of organic carbon and low level of contamination^21^. The wastewaters arise from various uses ranging from washing the sugar cane, melting, concentrating, filtering, purifying the sugar and washing the vessel and line. This wastewater contains high levels of organic carbons which are in most cases recycled to be used in molasses or yeast production systems^22^. However, the sugar refinery washing water (SRWW) which results from cleaning equipment and transfer lines are primarily used as an additive in cattle feeds. Therefore, the advantage of using SRWW for low-cost fermentation processes is apparent, though, careful characterization and optimization will be needed for application on specific target strains and products.

Generally, the productivity of certain metabolites based product is determined by the biomass productivity and the metabolites contents. While biomass productivity could be enhanced under favorable growth conditions, increasing the contents of some metabolites requires environmental stress. Therefore, various environmental factors have been applied as stimuli during cultivation in order to increase the contents of target metabolites^3,23,24^. It has been reported that lipid accumulation and composition are affected by environmental, chemical, and physical stimuli including salinity, growth medium pH, and temperature^25^. Among these, salinity is an important chemical stimulus causing physical osmotic pressure and commonly used to increase the lipid content of microalgae^26,27^. However, these factors are more often unfavorable for the growth of the organism and can reduce the overall biomass productivity. Taking these into account, the timing and level of the stimuli during cultivation also should be tested to maximize the productivity of the target metabolites by achieving the highest target product content when the cell biomass productivity was maximized^28^.

In this study, sugar refinery washing water (SRWW) was used as a carbon source for the production of DHA during heterotrophic cultivation of the marine microalga *Aurantiochytrium* sp. KRS101. To maximize the DHA yield, a serial optimization on culture nutrient and salt stress strategy were implemented. Firstly, the SRWW concentration and macronutrients (C:N:P) ratio were optimized to increase the biomass yield during the first stage of cultivation. Subsequently, sea salt stress was applied during the exponential or stationary phase to increase the DHA content. Finally, the effects of both the sea salt concentration and the timing of stress induction on the DHA content and yield were investigated.

## Results and Discussion

### Chemical properties of sugar refinery washing water (SRWW)

The growth profile and lipid productivity of *Aurantiochytrium* sp. KRS101 have been well studied using various carbon sources^29^. However, in the case of the undefined source such as SRWW, it is difficult to determine the nutrient consumption profile and substrate yield without proper characterization. Hence, in order to optimize *Aurantiochytrium* sp. KRS101 cultivation using SRWW as a sole carbon source, chemical and trace element properties of SRWW were analyzed (Table 1 and see Supplementary Material). The results revealed that there were sufficient concentrations of calcium (32 times higher), ferric (212 times higher), and magnesium (8 times higher) ions compared with the basal media (containing 10 g L^−1^ yeast extract), and the levels of other components were similar to those in conventional media. The initial pH of SRWW was 3.4, which is less than optimum for the cultivation of this strain^30^. Therefore, in subsequent experiments, the pH was adjusted to 7.0 using a 10 N NaOH solution. The total organic carbon (TOC) and chemical oxygen demand (COD) in SRWW were estimated to be 95,488 ± 3,818 and 295,600 ± 2,837 mg L^−1^, respectively. The glucose and sucrose concentrations in SRWW were measured separately and estimated to be 69,100 ± 930 and 5,050 ± 212 mg L^−1^, respectively. These results show that SRWW contained sufficient carbon sources to previous researches using 5 to 80 g L^−1^ of only glucose or combination with fructose, and sucrose for the heterotrophic cultivation of *Aurantiochytrium* sp. KRS101^31^. However, the concentrations of other essential nutrients, including nitrogen (ammonia-nitrogen: 2.34 mg L^−1^ and nitrate-nitrogen 27.15 mg L^−1^) and phosphorus (phosphate: 63.38 mg L^−1^), were relatively lower than previous research (ammonia-nitrogen: 246.40 mg L^−1^, nitrate-nitrogen: 246.40 mg L^−1^, phosphate: 105.00 mg L^−1^) or the basal medium (yeast extract: 10,000 mg L^−1^ and phosphate: 6,300 mg L^−1^) which is ordinarily used for *Aurantiochytrium* sp. KRS101 (Table 1)^30^. Thus in subsequent experiments, nitrogen and phosphorus were supplemented in the form of yeast extract and KH_2_PO_4_, respectively.

**Table 1:** Characteristics of the sugar refinery washing water (SRWW).

Parameter	Sugar refinery washing water (SRWW)
pH	3.40
Total organic carbon (TOC, mg L^−1^)	95,488 ± 3,818
Chemical oxygen demand (COD, mg L^−1^)	295,600 ± 2,837
Total carbohydrate (TC, mg L^−1^)	138,263 ± 867
Glucose (mg L^−1^)	69,100 ± 930
Sucrose (mg L^−1^)	5,050 ± 212
Ammonia-nitrogen (NH_4_-N, mg L^−1^)	2.387 ± 0.163
Nitrate-nitrogen (NO_3_-N, mg L^−1^)	27.147 ± 0.620
Nitrite-nitrogen (NO_2_-N, mg L^−1^)	ND
Phosphorus-phosphate (PO_4_-P, mg L^−1^)	63.377 ± 3.008

ND: not detected.

### Effect of SRWW dilution ratio on the heterotrophic cultivation of *Aurantiochytrium* sp. KRS101

**Figure 1:**
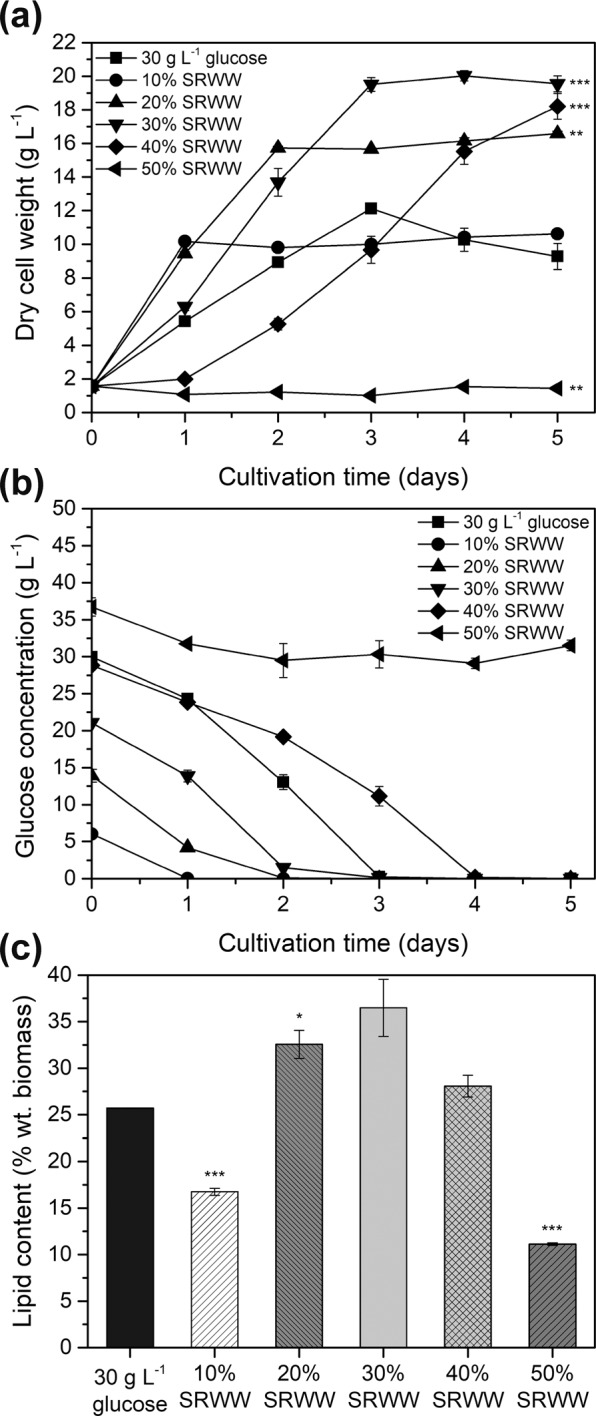
Effect of various concentrations of sugar refinery washing waster (SRWW) on the: (**a**) biomass yield (dry cell weight), (**b**) glucose concentration, and (**c**) lipid content of *Aurantiochytrium* sp. KRS101 cultured under heterotrophic conditions. Error bars indicate mean ± standard deviation (n = 6 for (**a**), and 4 for (**b**) and (**c**)). Statistical analysis was conducted with ANOVA tests (significant, P < 001) for (**a**) and (**c**) at day 5, and Student t-test, where ***P < 0.001, **P < 0.01, *P < 0.05 for all at day 5.

The effects of various concentrations of SRWW on growth, glucose concentration, and lipid production of *Aurantiochytrium* sp. KRS101 are shown in Fig. 1. Heterotrophic cultivation was tested using five dilutions of SRWW (10, 20, 30, 40, and 50%), and the growth parameters were compared with those achieved during cultivation in the modified basal media containing 30 g L^−1^ glucose (Fig. 1a). The biomass yield of *Aurantiochytrium* sp. KRS101 was measured as dry cell weight and it showed increased biomass yield as the concentration of SRWW increased (10.62, 16.59, and 19.55 g L^−1^, respectively, linear correlation (R^2^ = 0.9636)) from 10 to 30% at day 5. However, growth was inhibited when the SRWW concentration was higher than or equal to 40%, and there was no growth at 50% SRWW indicating that there was an optimal concentration of SRWW suitable for heterotrophic cultivation. Several studies have shown that excessive nutrients in growth media cause osmotic stress, which inhibits algal growth^32,33^. This is the main reason why high-density heterotrophic cultivation is often performed in continuous or semi-continuous fed-batch mode rather than with all the carbon source added to the medium at the start of cultivation. Consistent with previous research^34^, it appeared that excessive glucose at the commencement of culture caused osmotic shock to *Aurantiochytrium* cells, as cell disruption was observed when the cells were exposed to 50% SRWW. When the SRWW concentration was increased further (up to 60%), it was rare to find any normal cells in the culture liquid (see Supplementary Material). The concentration of glucose declined rapidly during cultivation, except in the treatment containing 50% SRWW, where no growth occurred (Fig. 1b).

Here, it should be noted that although the 30% SRWW medium contained less glucose (20 g L^−1^) than modified basal medium (30 g L^−1^), the maximum biomass yield achieved was greater in the former medium. One explanation for this observation is the presence of other carbon sources in SRWW. As sucrose can be disassociated into glucose and fructose under low pH conditions (Table 1)^35^, there were additional amounts of glucose and fructose that were not accounted for. A previous study also reported that *Aurantiochytrium* sp. KRS101 is capable of using various organic carbon sources^36^. Further studies will be needed to assess the possibility of carbon catabolite repression and the effects of multiple sugars in growth media during heterotrophic cultivation of *Aurantiochytrium* sp. KRS101.

Another important factor in industrial fermentation is the conversion ratio from the glucose feedstock into cell biomass. As the SRWW concentration in the medium was increased (10, 20, 30, and 40%), the glucose to biomass conversion ratio declined (1.13, 0.97, 0.82, and 0.55 g biomass g^−1^ glucose, respectively). However, if the fructose converted from all the sucrose in the medium is taken into account, the total sugar to biomass conversion ratio for 40% SRWW would be 0.28, which is comparable to that in the basal medium control (Fig. 1b).

The lipid content was also affected by the concentration of SRWW (Fig. 1c) and showed a proportional correlation with the biomass yield (Fig. 1a, linear correlation (R^2^ = 0.9797)) when 10 to 30% SRWWs were used (30% SRWW - 19.55 g L^−1^ and 36.48% wt. biomass, 20% SRWW - 16.59 g L^−1^ and 32.56% wt. biomass, and 10% SRWW - 10.62 g L^−1^ and 16.74% wt. biomass). Among the tested conditions, the highest biomass yield (19.55 g L^−1^ at 5 days) and lipid content (36.48% wt. biomass) were achieved at 30% SRWW. In the control medium (30 g L^−1^ glucose), the cells entered death phase immediately after all the glucose in the medium was depleted; the biomass yield and lipid content in the control were 9.28 g L^−1^ in 5 days and 25.70% wt. biomass, respectively. These results confirmed that SRWW could be used as an alternative carbon source for the heterotrophic cultivation of *Aurantiochytrium* sp. KRS 101, with no negative effect on the lipid content. Consequently, 30% SRWW was used in subsequent experiments.

### Effect of the concentrations of KH_2_PO_4_ and yeast extract on cell growth and lipid production

**Figure 2:**
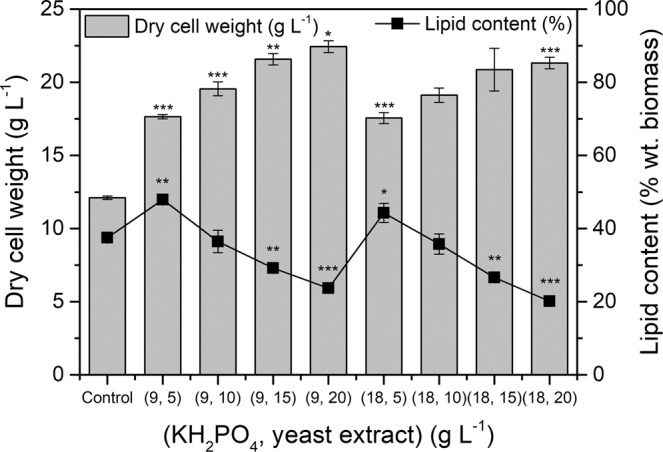
Changes in biomass yield (dry cell weight) (gray bars) and lipid content (black squares) of *Aurantiochytrium* sp. KRS101 using 30% SRWW supplemented with various concentrations of yeast extract and KH_2_PO_4_. The control involved the culture grown in conventional modified basal media containing 30 g L^−1^ glucose. Two concentrations of KH_2_PO_4_ (9 and 18 g L^−1^) and four concentrations of yeast extract (5, 10, 15, and 20 g L^−1^) were tested. Error bars indicate mean ± standard deviation (n = 3 and 6). ANOVA tests were conducted for biomass yield and lipid content, and both showed significant differences (P < 0.001). ANOVA test with post-hoc Tukey Honestly Significant Difference showed that groups having same concentration of phosphate ((9.5)–(18, 5), (9, 10)–(18, 10), (9, 15)–(18, 15), and (9, 20)–(18, 20)) have no significant differences (P > 0.05) for biomass yield and lipid content. Student t-test was also conducted and showed significant differences with control as ***P < 0.001, **P < 0.01, *P < 0.05.

The effects of nutrient conditions on cell growth and lipid production were investigated by testing various concentrations of nitrogen (yeast extract: 5, 10, 15, and 20 g L^−1^) and phosphorus (KH_2_PO_4_: 9 and 18 g L^−1^) in the medium (Fig. 2). There was a negligible difference in the biomass yield and lipid content between media containing 9 and 18 g L^−1^ KH_2_PO_4_; this is because 9 g L^−1^ exceeded the minimum phosphorus requirement for growth. The concentration of phosphorus in organisms is far less than that of carbon or nitrogen; the Redfield ratio for C, N, and P in oceanic plankton is 106:16:1^37^. To prepare for prolonged periods of exposure to conditions where phosphorus may be limiting, microalgae continuously uptake phosphorus and store the excess in the form of intracellular phosphate granules^38^.

In contrast to phosphorus, the nitrogen content affected both the biomass yield and the lipid content (Fig. 2). The proportion of nitrogen in cell mass is relatively high compared with that of phosphorus and is directly linked to the *de novo* synthesis of proteins responsible for growth, metabolism, and other biological processes. Therefore, cultures supplied with higher levels of yeast extract showed higher biomass yield but had lower lipid content levels. These findings are consistent with previous research^31^, which showed that increased nitrogen concentrations had a negative relationship with lipid accumulation. In addition, a previous study^30^ showed that the C:N ratio is a critical factor determining lipid accumulation in cells. As the microalgae had very low C:N ratios under the higher nitrogen concentrations tested, it is likely that higher lipid content and productivity could be achieved by feeding an additional carbon source.

The maximum biomass yield (22.44 g L^−1^ in 5 days) was obtained in the medium containing 9 g L^−1^ KH_2_PO_4_ and 20 g L^−1^ yeast extract, but the highest lipid content (47.93% wt. biomass) was obtained in the medium containing 9 g L^−1^ KH_2_PO_4_ and 5 g L^−1^ yeast extract. Thus, the optimum growth condition for the first stage of cultivation was determined to be 9 g L^−1^ KH_2_PO_4_ and 20 g L^−1^ yeast extract based on the biomass yield.

### Effect of salinity stress on biomass and lipid accumulation of *Aurantiochytrium* sp. KRS101

**Figure 3:**
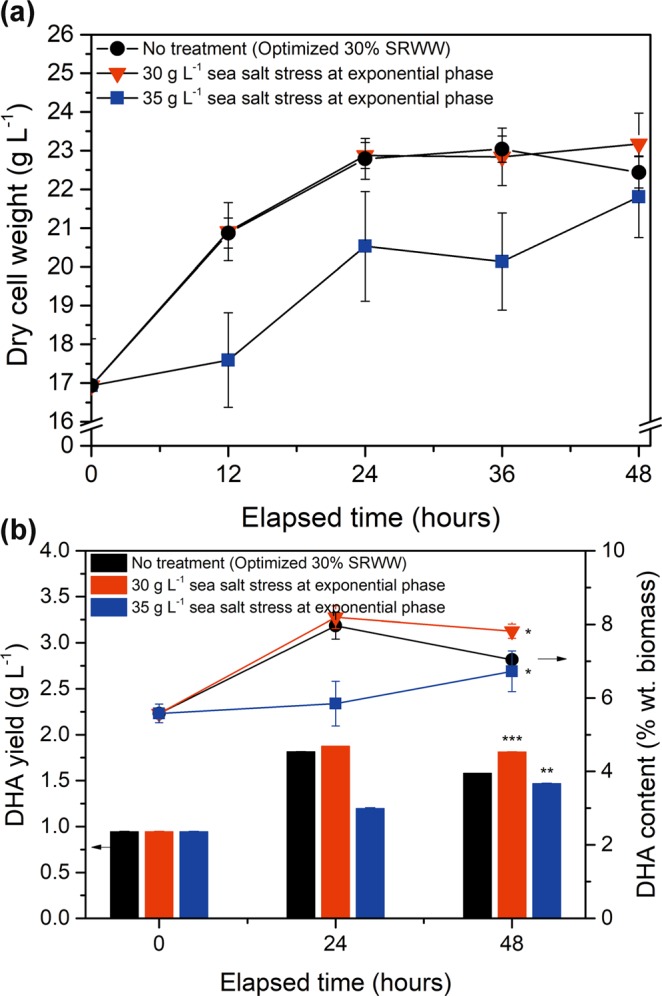
Effect of various concentrations of sea salt on the: (**a**) biomass yield (dry cell weight), (**b**) DHA yield (bar), and DHA content (line) of *Aurantiochytrium* sp. KRS101 cells in exponential phase. Error bars indicate mean ± standard deviation (n = 3). Statistical analysis were conducted with ANOVA tests for (**a**) and (**b**) at 48 h, and (**a**) at 48 h has no significant differences (P > 0.05) and DHA yield and DHA content at 48 h have significant differences as P < 0.001 and P < 0.01, respectively. Also, Student t-test was conducted and showed significant differences with no treatment as ***P < 0.001, **P < 0.01, *P < 0.05.

**Figure 4:**
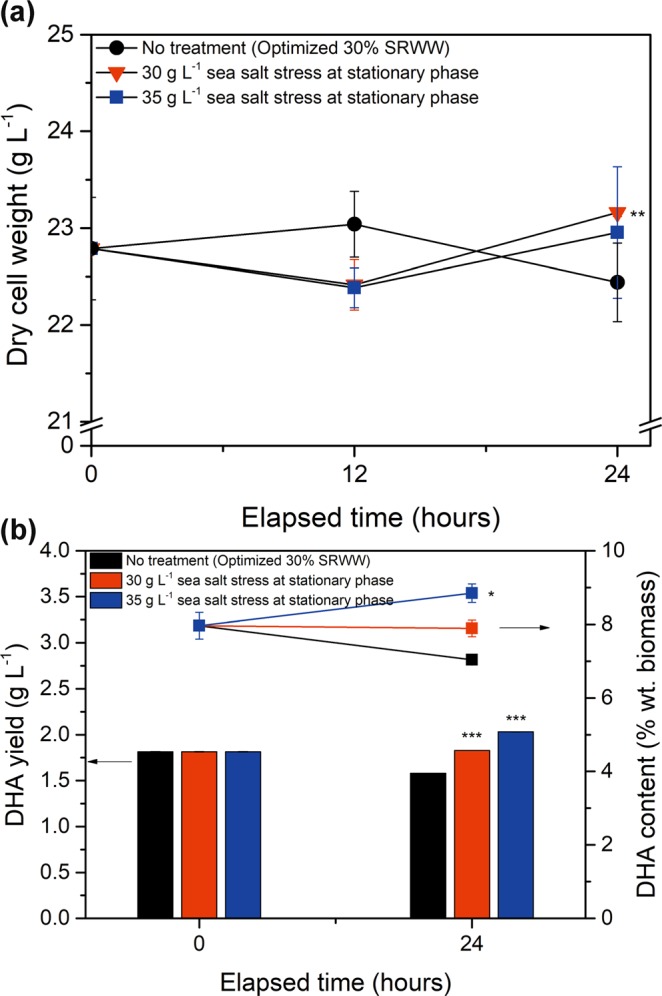
Effect of various concentrations of sea salt on the: (**a**) biomass yield (dry cell weight), (**b**) DHA yield (bar), and DHA content (line) of *Aurantiochytrium* sp. KRS101 cells in stationary phase. Error bars indicate mean ± standard deviation (n = 3). ANOVA tests were conducted for (**a**) and (**b**) at 24 h, and (**a**) at 24 h showed no significant differences (P ≈ 0.05) and DHA yield and DHA content have significant differences as P < 0.001 and P < 0.05, respectively. Also, Student t-test was conducted and showed significant differences with no treatment as ***P < 0.001, **P < 0.01, *P < 0.05.

Optimization of the SRWW concentration as well as the levels of KH_2_PO_4_ and yeast extract successfully increased the overall biomass production (Fig. 2). However, the increase in biomass yield was accompanied by a decrease in the lipid content, because the C:N ratio was suboptimal for lipid content. To attempt to compensate for this loss, the effect of sea salt stress on DHA accumulation in *Aurantiochytrium* sp. KRS101 was investigated. The effects of higher sea salt concentrations (30 and 35 g L^−1^) during the exponential (Fig. 3) and stationary (Fig. 4) phases on DHA yields, were investigated. It should be noted that the cells underwent 24 and 48 hours of osmotic stress when the stress was implemented on exponential and stationary phases, respectively.

When the culture was treated with 35 g L^−1^ of additional sea salt during the exponential phase, growth suppression was observed after 12 h, although gradual recovery occurred towards the end of the cultivation period (48 h; Fig. 3a). Interestingly, however, the addition of 30 g L^−1^ sea salt resulted in an almost identical growth curve to that of the control indicating that the sea salt concentration did not cause osmotic stress. In addition, a slight increase in the overall DHA content was observed with 30 g L^−1^ of osmotic stress, while a small decrease was observed with 35 g L^−1^. Subjecting the cells to 30 g L^−1^ of salt stress resulted in an increase in the DHA yield, but 35 g L^−1^ of additional salt. The utilization the overall yield because of the combination of lower biomass production and lower DHA content per unit biomass. In general, it appeared that subjecting the cells to osmotic stress during the exponential phase did not result in a substantial increase in DHA production. It is likely due to the fact that the culture was bottlenecked by the lack of carbon availability, and particularly that the higher nitrogen content during the first stage of growth resulted in greater cell proliferation. Thus, the cells were unable to assimilate extra carbon from the environment for *de novo* lipid synthesis, despite being under osmotic stress.

When additional sea salt was added at the stationary phase there was no significant change in biomass yield under any tested conditions (Fig. 4a). This is probably because the cells had assimilated all the nutrients during the first stage of growth. The DHA content in the control culture showed a minor decrease, from 7.96 ± 0.36% wt. biomass at the start of the stationary phase to 7.04 ± 0.06% wt. biomass after 24 h. However, cultures under osmotic stress showed an increase in the DHA content with 30 g L^−1^ and 35 g L^−1^ of additional sea salt yielding DHA contents of 7.89 ± 0.23% wt. biomass and 8.85 ± 0.25% wt. biomass, respectively (Fig. 4b). The increase in the DHA content also resulted in an improvement in the DHA yield under both the 30 and 35 g L^−1^ treated osmotic stress conditions. Therefore, these results indicated that subjecting the cells to osmotic stress at the beginning of the stationary phase was more effective at improving DHA production than applying the stress during the exponential phase. Though DHA content in stationary phase treatment looked similar with that in exponential phase treatment, it is coupled with stable biomass yield and enhanced lipid content, and resulted in the highest DHA yield with 35 g L^−1^ sea salt treatment at day 5 as Fig. 4b.

Previous studies have reported that *Aurantiochytrium* sp. is insensitive to the sea salt concentration during the initial phase of growth^31,39^. In contrast, these results revealed that treating the culture with higher concentrations of sea salt during the exponential phase caused some reduction in biomass production, while osmotic stress applied during the stationary phase resulted in higher DHA production with little negative impact on the biomass. However, it should be noted that the study was unable to exploit the full potential of the sea salt stress, due to the limitations on *de novo* lipid synthesis imposed upon by low C:N ratio.

### Comparison of biomass yield, lipid yield, DHA yield, and the fatty acid profile of cells under various culture conditions

**Figure 5:**
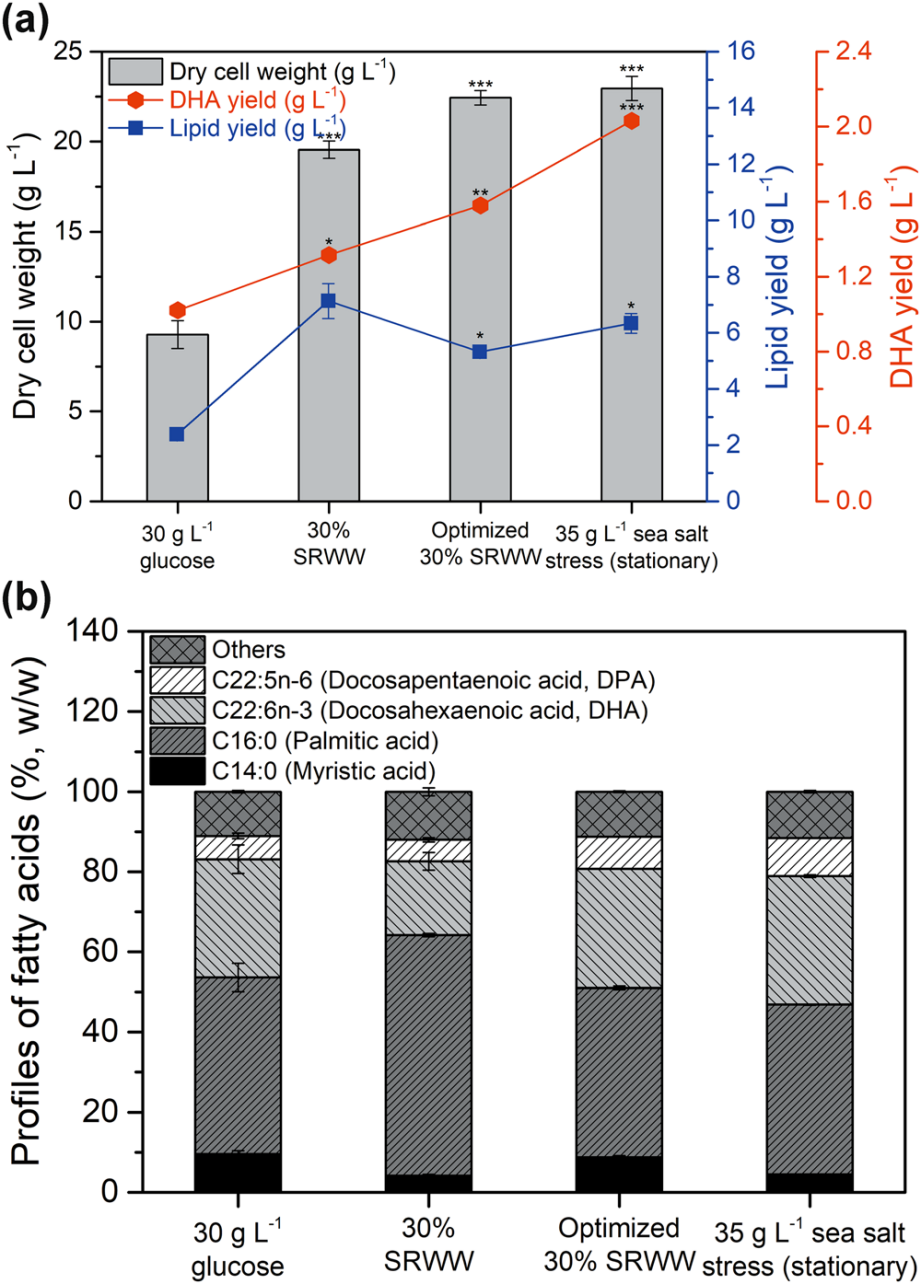
Changes in the: (**a**) biomass yield (dry cell weight, gray bars), lipid yield (blue squares), DHA yield (red circles), and (**b**) fatty acid profiles of *Aurantiochytrium* sp. KRS101 cultured under various heterotrophic conditions of sea salt stress. Error bars indicate mean ± standard deviation (n = 3). ANOVA tests were conducted for (**a**) and it showed significant differences (P < 0.001) for all biomass yield (dry cell weight), lipid yield, and DHA yield. Also, Student t-test was conducted and showed significant differences with no treatment as ***P < 0.001, **P < 0.01, *P < 0.05.

To assess the effects of SRWW concentration, nutrient optimization, and stress induction on the biomass, lipid, and DHA yields, the fatty acid profiles of *Aurantiochytrium* sp. KRS101 were compared as shown in Fig. 5. Use of 30% SRWW significantly increased the biomass yield, lipid yield, and DHA yield (biomass yield (DCW): 9.27 to 19.55 g L^−1^; lipid yield: 2.38 to 7.13 g L^−1^; DHA yield: 1.02 to 1.31 g L^−1^) of *Aurantiochytrium* sp. KRS101 (Fig. 5a), mainly because of the enhanced DCW and lipid content. The total DHA yield also increased, despite the fact that the DHA composition in the fatty acid profile decreased from 29.50 to 25.66% (Fig. 5b). It appears that the higher TOC level in the 30% SRWW enabled the microalgae to achieve higher biomass yields as well as lipid content. Substitution of glucose with SRWW also caused changes in the fatty acids profile that there was increase in the portion of saturated fatty acids (SFAs) and it was mainly caused by a shift from C14:0 (9.54, 4.18%, respectively) to C16:0 (44.09, 59.96%, respectively) and a decrease in the portion of one of the polyunsaturated fatty acids (PUFAs, especially DHA; C22:6 n-3). In the previous research^19^, similar sugar factory wastewater was used as an alternative carbon source on green algae, *Ettlia* sp., and it showed decrease in the portion of both SFAs and PUFAs, and increase in the portion of monounsaturated fatty acids (MUFAs). The utilization of carbon sources from both SRWW and sugar factory wastewater showed better cell growth and lipid accumulation in both cases, but differences in a genus and specific culture conditions caused verified changes in fatty acids profile. Additional optimization of the nitrogen and phosphorus sources further enhanced the performance of the 30% SRWW, as the total DCW increased up to 22.44 g L^−1^. Despite the decrease lipid content (30% SRWW - 36.48 to optimized 30% SRWW - 23.71%wt. biomass, Fig. 2) caused by excessive nutrients, the increase in the DHA content in the lipid (30% SRWW - 18.45 to 35 g L^−1^ sea salt stress - 32.04%wt. fatty acids, Fig. 5b) resulted in a marked improvement in the DHA yield (up to 1.57 g L^−1^). The DHA yield was also improved (up to 2.03 g L^−1^) by subjecting stationary phase cells to sea salt stress. Therefore, adopting 30% SRWW is successfully increase the biomass yield, optimized nutrient condition stabilize DHA content, and sea salt stress further enhanced DHA content.

## Conclusion

SRWW containing high levels of organic carbon in the form of saccharides was applied to the heterotrophic cultivation of *Aurantiochytrium* sp. KRS101. Compared with the general basal medium, cultivation in optimized 30% SRWW resulted in higher biomass (2.41-fold increase), lipid content (2.23-fold higher), and DHA yield (1.54-fold increase). In addition, the total DHA yield was improved by 28% when the cells were subjected under sea salt stress by supplementation of the medium with 35 g L^−1^ additional sea salt at the stationary stage. It is likely that the overall lipid content and DHA production could be further improved by increasing the C:N ratio.

## Methods

### Microalgal strain and inoculum preparation

The marine heterotroph *Aurantiochytrium* sp. KRS101 was kindly provided by Dr. Jeong-Woo Seo (Korea Research Institute of Bioscience and Biotechnology, Jeongup, Korea; KRIBB). Cells were cultivated until cell density obtained to 10 g L^−1^ with basal medium (containing 60 g L^−1^ glucose, 15 g L^−1^ sea salt, 10 g L^−1^ yeast extract (70161; Sigma Aldrich, USA), 9 g L^−1^ KH_2_PO_4_, and 10 mg L^−1^ tetracycline) at 120 rpm and 25 ± 1 °C with 200 mL working volume in a dark incubating shaker^31^. Then, cells were harvested by centrifugation at 6,523 × g (8000 rpm, Supra R22; Hanil Science, Korea) for 5 min and resuspended to have 20 g L^−1^ cell density with fresh basal medium containing 25% (v/v) glycerol and the aliquoted (2 mL) cell stocks were stored at −80 °C. Seed cultures were prepared with 2 mL of cell stock and cultivated for 3 days in 250 mL baffled flasks (DURAN®) containing 100 mL basal medium using in a dark incubating shaker operating at 120 rpm and 25 ± 1 °C, and were subsequently used for inoculations.

### Preparation of sugar refinery washing water (SRWW)

The SRWW used in this study was collected from a local sugar manufacturing facility in Korea. Before use in experiments, in order to avoid clumps and aggregation of suspended solids, they were naturally settled down at room temperature overnight and removed. After that, additional particles were centrifuged at 6,523 × g (8000 rpm, Supra R22; Hanil Science, Korea) for 10 min. The supernatant was filtered through a 0.22 μm bottle top filtration device (Sartolab® Vacuum Filtration devices, Sartorius Stedim Biotech; Göttingen, Germany) and stored in a 20 L plastic container at 4 °C. The nutrients (carbon, nitrogen, and phosphate sources) available for microalgal cultivation in the SRWW were characterized using previously reported procedures^19^. Based on the characterization information, the pH was adjusted to 7.0 where the best DHA yield was achieved using 10 N NaOH^30^, and various amounts of yeast extract and KH_2_PO_4_ were supplemented according to the experimental protocol.

### Heterotrophic cultivation conditions

*Aurantiochytrium* sp. KRS101 cells were grown in 500 mL baffled flasks (DURAN®) containing 200 mL of modified basal medium (30 g L^−1^ glucose, 15 g L^−1^ sea salt, 10 g L^−1^ yeast extract (70161; Sigma Aldrich, USA), 9 g L^−1^ KH_2_PO_4_, and 10 mg L^−1^ tetracycline) as a medium control, or in SRWW medium (supplemented with 15 g L^−1^ sea salt, 10 g L^−1^ yeast extract (70161; Sigma Aldrich, USA), 9 g L^−1^ KH_2_PO_4_, and 10 mg L^−1^ tetracycline) in an dark incubating shaker operating at 200 rpm and 25 ± 1 °C. To investigate the effect of SRWW on heterotrophic cultivation of *Aurantiochytrium* sp. KRS101, five concentrations of SRWW (10, 20, 30, 40, and 50%) were tested in place of glucose in the modified basal medium (30 g L^−1^ glucose). To evaluate the effects of varying combinations of yeast extract and KH_2_PO_4_ on the biomass yield of *Aurantiochytrium* sp. KRS101, four concentrations (5, 10, 15, and 20 g L^−1^) of yeast extract and two concentrations (9 and 18 g L^−1^) of KH_2_PO_4_ were supplemented into a 30% SRWW medium (which was confirmed as an optimum dilution rate) containing 15 g L^−1^ sea salt and 10 mg L^−1^ tetracycline. The initial cell density was 1.5 g L^−1^ (dry cell-based) and the cultivation period was 5 days.

The influence of sea salt stress on DHA production was studied using various concentrations of sea salt, duration of stress, and cell growth stages at the time of exposure. *Aurantiochytrium* sp. KRS101 cells were grown in 500 mL baffled flasks (DURAN®) containing 200 mL of the optimized SRWW medium (30% SRWW, 9 g L^−1^ KH_2_PO_4_, 20 g L^−1^ yeast extract (70161; Sigma Aldrich, USA), 15 g L^−1^ sea salt, and 10 mg L^−1^ tetracycline). Additional sea salt was added into the flask containing the optimized SRWW medium during the exponential (3rd day; 30 g L^−1^) or stationary (4th day; 35 g L^−1^) growth phases, resulting in the cells being exposed to sea salt stress for 2 days and 1 day, respectively. The optimum SRWW medium without sea salt supplementation was used as a control in this experiment. The cells were harvested every 12–24 h following sea salt supplementation, for analysis of the biomass and lipid content.

### SRWW characterization

Total organic carbon (TOC) and total carbon (TC) were determined using a TOC analyzer (TOC-V; Shimadzu, Kyoto, Japan). Chemical oxygen demand (COD) was measured using a water test kit (HS-COD-MR; Humas, Korea) and UV-spectrometer (DR2010; Hach, Loveland, CO, USA). Nutrients (ammonium, nitrate, nitrite, and phosphate) were analyzed using ion chromatography (883 Basic IC plus, Metrohm AG, Herisau, Switzerland); a Metrosep A Supp 5- 150/4.0 (Metrohm AG; Herisau, Switzerland) column was used for anion analysis and a Metrosep C4-150/4.0 (Metrohm AG; Herisau, Switzerland) was used for cation analysis. Trace elements were determined using an inductively coupled plasma optical emission spectrometry (ICP-OES) on a Perkin-Elmer Optima 3300 DV spectrometer.

### Dry cell weight, residual saccharide analysis, and cell imaging

Cell growth was solely measured with dry cell weight because dry cell weight represents more accurate cell growth than cell number in this higher cell density fermentation. Equal volumes of microalgal culture (10 mL) were harvested by centrifugation at 6,523 × g (8000 rpm, Supra R22; Hanil Science, Korea) for 5 min and washed twice with phosphate-buffered saline (PBS, pH 7.4) to remove micronutrient from the media. This PBS buffer was used to avoid cell disruption during the harvesting^30^ and most of cell density was higher than 10 g L^−1^, therefore the effect of salt in the PBS buffer is negligible. The supernatant was filtered through a 0.20 μm syringe filter (16534, Sartorius Stedim Biotech; Göttingen, Germany) prior to analysis using a YSI glucose analyzer (YSI 2900; YSI Life Science, OH, USA) to determine the residual saccharide concentration. The cell pellets were freeze-dried for 3 days in pre-weighed tubes, and the dry cell weight was calculated based on weight difference. Microscopic observations were carried out at 100× magnification using inverted optical microscopy (DM2500; Leica, Germany), and images were recorded using a DFC425C Leica microscope camera.

### Fatty acid methyl esters (FAMEs) analysis

Fatty acid methyl esters (FAMEs) were analyzed using gas chromatography (GC), as described previously^40^. Total lipids from 10 mg of lyophilized biomass were extracted by vortexing for 10 min in 2 mL of a 2:1 chloroform−methanol mixture. A chloroform solution (1 mL) containing 0.5 mg of nonadecanoic acid (C19:0) was added as an internal standard, and 1 mL of methanol and 300 μL of sulfuric acid were added for the transesterification reaction, which occurred at 100 °C for 20 min. After all intracellular lipids were converted to FAMEs by transesterification, deionized water (1 mL) was added to wash out residual methanol and sulfuric acid. The samples were then centrifuged at 1,631 × g (4000 rpm, Supra R22; Hanil Science, Korea) for 10 min to separate the organic and aqueous phases. The FAMEs in the organic phase were identified based on the retention time using FAMEs standard (47885-U, Supelco® FAME Mix, USA) and quantified with internal standard (nonadecanoic acid, C19:0) by GC (HP6890; Agilent, USA) using a flame ionized detector (FID) and an INNOWAX capillary column (30 m × 0.32 mm × 0.5 μm; Agilent, USA). The initial temperature was 50 °C (held for 1 min) and it increased with a rate of 15 °C min^−1^ to 200 °C (held for 9 min) and it increased again with a rate of 2 °C min^−1^ to 250 °C (held for 2 min). DHA which is one of the FAMEs was identified, quantified, and calculated as DHA yield (g DHA L^−1^ used medium) and DHA content (% g DHA g^−1^ biomass).

### Statistical analysis

All the experiments were conducted at least 3 times of biological replication and 2 times of technical replication. One-way analysis of variance (ANOVA) was conducted to test the statistical difference of the related parameters of each condition using Excel. Also, one-way ANOVA with post-hoc Tukey Honestly Significant Difference (HSD) was applied to check the certain groups having no significant difference among all groups using Excel.

## Supplementary information

Supplementary Information
