# Supplementary material for: Biological Carbon Recovery from Sugar Refinery Washing Water into Microalgal DHA: Medium Optimization and Stress Induction
Source: Sci Rep. 2019 Dec 27;9:19959. doi: 10.1038/s41598-019-56406-x (PMC6934592; doi:10.1038/s41598-019-56406-x)

Biological Carbon Recovery from Sugar Refinery Washing Water into Microalgal DHA: Medium Optimization and Stress Induction

Myounghoon Moon[1, +], Won-Kun Park[2, +], William I. Suh[3], Yong Keun Chang [3, 4, *], Bongsoo Lee[5, *]

[1] Gwangju Bio/Energy R&D Center, Korea Institute of Energy Research (KIER), 25, Samso-ro 270beon-gil, Buk-gu, Gwangju, 61003, Republic of Korea

[2] Department of Chemistry & Energy Engineering, Sangmyung University, 20 Hongjimun 2-gil, Jongno-gu, Seoul, 03016, Republic of Korea

[3] Advanced Biomass R&D Center, Korea Advanced Institute of Science & Technology (KAIST), 291 Daehak-ro, Yuseong-gu, Daejeon 34141, Republic of Korea

[4] Department of Chemical and Biomolecular Engineering, Korea Advanced Institute of Science & Technology (KAIST), 291 Daehak-ro, Yuseong-gu, Daejeon, 34141, Republic of Korea

[5] Department of Microbial and Nano Materials, College of Science and Technology, Mokwon University, 88 Doanbuk-ro, Seo-Gu, Daejeon, 35349, Republic of Korea

[+] Myounghoon Moon and Won-Kun Park contributed equally to this work.

[*]Corresponding authors

E-mail address: bongsoolee@mokwon.ac.kr (B. Lee); ychang@kaist.ac.kr (Y.K. Chang)

Phone numbers: +82-42-829-7555 (B. Lee); +82-42-350-3927 (Y.K. Chang)

Supplementary table and figure

Table S1: Trace element analysis of SRWW (sugar refinery washing water) using ICP-OES (Inductively coupled plasma optical emission spectrometry).

**Trace elements**	**SRWW, mg L^{-1}**
Al	0.93 ± 0.00
Ca	101.60 ± 0.62
Fe	85.10 ± 1.05
K	228.22 ± 2.14
Li	< 0.1
Mg	18.41 ± 0.11
Mn	0.60 ± 0.02
Cd	< 0.1
Cu	< 0.1
Ni	< 0.1
Pb	< 0.1
Zn	0.98 ± 0.02
Cr	< 0.1

Figure S1: Differential interference contrast (DIC) micrographs of *Aurantiochytrium* sp. KRS101 cells in heterotrophic cultivation using basal medium containing SRWW (sugar refinery washing water). (A) 60% SRWW and (B) 50% SRWW. The scale bar is 10 μm.

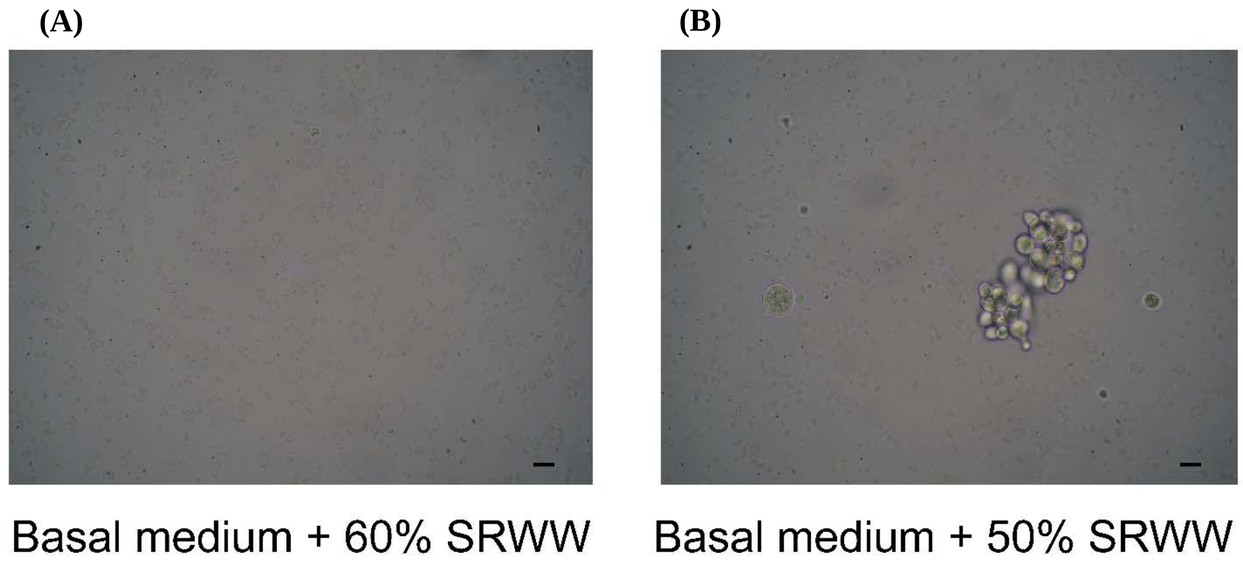

Supplement: Supplementary file 1 — Supplementary Information [file 41598_2019_56406_MOESM1_ESM.pdf]
